# PP101 A Review Of Health State Utility Values Associated With Herpes Zoster

**DOI:** 10.1017/S0266462324002630

**Published:** 2025-01-07

**Authors:** Aoife Bergin, Helen O’Donnell, Susan Ahern, Joan Quigley, Patricia Harrington, Conor Teljeur, Máirín Ryan

## Abstract

**Introduction:**

Herpes zoster and post-herpetic neuralgia (PHN) can cause significant pain and morbidity, impacting on the health-related quality of life of those affected. This research aimed to identify primary studies that elicited health state utility values (HSUVs) relating to both acute herpes zoster and PHN, and to assess their suitability for an economic model of herpes zoster vaccination.

**Methods:**

Cost-effectiveness analyses of herpes zoster vaccination were identified from (i) a recently published systematic review of economic evaluations of herpes zoster vaccination and (ii) an independently conducted rapid review that sought to update the results of the identified systematic review. The evaluations were hand-searched to identify original studies that elicited HSUVs of interest. Narrative synthesis of the original study characteristics was conducted and possible sources of variation in the studies identified. Appraisal of the methodological quality of the studies was undertaken using a published open response tool that outlined key quality assessment criteria for HSUV studies.

**Results:**

Twenty-one studies that elicited HSUVs were identified. Values related either to changes in utility resulting from increased pain severity, or to changes with time since disease onset. Heterogeneity was observed, potentially caused by differences in population size and characteristics, methods of elicitation, and preference weights used to value utility. Methodological quality varied across studies, with concerns around low sample sizes for populations with PHN, and poor reporting of both missing data and uncertainty around the HSUVs obtained. Differences in study population, time of participant recruitment, and methods of measuring utility could limit the applicability of HSUV study data.

**Conclusions:**

This study highlights both the heterogeneity in HSUVs obtained for herpes zoster and the challenges associated with selecting HSUV data suitable for an economic model. An evidence base from which to select values for an economic evaluation has been created, though poor methodological quality and reporting of the primary studies may compromise the validity of point estimates obtained.

